# Human skin dermis-derived fibroblasts are a kind of functional mesenchymal stromal cells: judgements from surface markers, biological characteristics, to therapeutic efficacy

**DOI:** 10.1186/s13578-022-00842-2

**Published:** 2022-07-12

**Authors:** Chenxu Tai, Zhengyao Xie, Yu Li, Yirui Feng, Yuanyuan Xie, Hui Yang, Liudi Wang, Bin Wang

**Affiliations:** 1 Clinical Stem Cell Center, The Affiliated Drum Tower Hospital of Nanjing University Medical School, Nanjing, 210008 People’s Republic of China; 2 National Clinical Research Center of Kidney Diseases, Jinling Hospital, Nanjing University School of Medicine, Nanjing, 210016 People’s Republic of China; 3 State Key Laboratory of Pharmaceutical Biotechnology and The Comprehensive Cancer Center, The Affiliated Drum Tower Hospital of Nanjing University Medical School, School of Life Science, Nanjing University, Nanjing, Jiangsu China

**Keywords:** Cell therapy, Fibroblasts, Mesenchymal stromal cells, Hepatic fibrosis, Colitis

## Abstract

**Background:**

Human mesenchymal stromal cells (MSCs) have been widely advocated to clinical use. Human skin dermis-derived fibroblasts shared similar cellular morphology and biological characteristics to MSCs, while it still keeps elusive whether fibroblasts are functionally equivalent to MSCs for therapeutic use.

**Methods:**

We isolated various fibroblasts derived from human foreskins (HFFs) and human double-fold eyelids (HDF) and MSCs derived from human umbilical cords (UC-MSCs), and then comprehensively investigated their similarities and differences in morphology, surface markers, immunoregulation, multilineage differentiation, transcriptome sequencing, and metabolomics, and therapeutic efficacies in treating 2,4,6-Trinitrobenzenesulfonic acid (TNBS) induced colitis and carbontetrachloride (CCL_4_) induced liver fibrosis.

**Results:**

Fibroblasts and UC-MSCs shared similar surface markers, strong multilineage differentiation capacity, ability of inhibiting Th1/Th17 differentiation and promoting Treg differentiation in vitro, great similarities in mRNA expression profile and metabolites, and nearly equivalent therapeutic efficacy on TNBS-induced colitis and CCL_4_-induced hepatic fibrosis.

**Conclusion:**

Human skin dermis-derived fibroblasts were a kind of functional MSCs with functionally equivalent therapeutic efficacy in treating specific complications, indicating fibroblasts potentially had the same lineage hierarchy of origin as MSCs and had a remarkable potential as an alternative to MSCs in the treatment of a variety of diseases.

**Supplementary Information:**

The online version contains supplementary material available at 10.1186/s13578-022-00842-2.

## Introduction

Since the concept of bone marrow mesenchymal stromal cells (BM-MSCs) was first proposed in 1968 [1, 2], various tissue-derived MSCs such as umbilical cords, placentas, and pulp of teeth have been extensively advocated to the treatments of type 1 diabetes [3], systemic lupus erythematosus [4], rheumatoid arthritis [5], hepatic fibrosis [6], and Crohn's disease [7] due to their multilineage differentiation potentials and immunoregulatory capability [8, 9].

There is evident heterogeneity among these MSCs isolated from different tissues. In order to define MSCs, the International Society for Cellular Therapy (ISCT) proposed a set of minimum standards for cell therapy including plastic adhesion under standard culture conditions, negatively expressing CD14, CD19, CD34, CD45, and HLA-DR, as well as highly expressing CD73, CD90, and CD105, maintainingmultilineage differentiation potentials of osteogenesis, chondrogenesis, and adipogenesis in vitro [10].

However, the definition of MSCs has been controversial for several decades, and MSCs were still elusive. In fact, the surface markers used to define MSCs were also found in fibroblasts [11]. Fibroblasts as one major type of stromal cells, deposit the collagen and other components of the extracellular matrix (ECM) in connective tissues. As a group of heterogeneous cells from a wide range of sources, fibroblasts can derive from different parts of skin dermis, and participate in scar formation and heeling of skin injury or burning [12–14]. Actually, researches on the comparisons between human MSCs and fibroblasts have been carried on over 30 years. Cappeleso-fleurie et al. [15] reported that MSCs differed from dermal and foreskin fibroblasts due to their differentiation ability with different expression levels of CD10, CD106, and CD26. Wagner et al. found that fibroblasts derived from human foreskin had different multilineage differentiation capabilities from MSCs and could not differentiate into lipoblast or osteoblast cells [16]. However, in recent years, it has been reported that fibroblasts exhibited more and more characteristics of MSCs such as low immunogenicity, multilineage differentiation ability, and immunoregulatory capability in vitro [11, 15]. The immunosuppressive property was considered a key feature of MSCs [17]. It was widely believed fibroblasts had no similar immunosuppressive effect to MSCs [10]. However, accumulating ontological studies have suggested that fibroblasts have interaction with immune system acting as alternative antigen resenting cells (APC) by down-regulating or activating T cells [18–20] and in directly regulating the anti-proliferation of lymphocytes [21, 22].

Now that fibroblasts have been found to have all characteristics used to define MSCs, but these researches are fragmented and focus on one or more specific aspects of biological features of MSCs and fibroblasts. There is little study regarding whether fibroblasts also have the same therapeutic properties with MSCs in various disorders. In this study, we performed high throughput transcriptome sequencing and metabolomics analysis to investigate the intrinsic natures of human umbilical cord-derived MSCs and skin dermis-derived fibroblasts. In addition, we also compared the therapeutic efficacy between MSCs and fibroblasts on colitis and hepatic fibrosis models. The results showed that human fibroblasts are a kind of functional MSCs, sharing almost same intrinsic natures as MSCs including surface markers, biological characteristics, and therapeutic efficacy in treating specific complications. Our study provided us further understanding of MSCs and fibroblasts and indicated that fibroblasts had a remarkable potential as alternative cells for MSCs in treatments of a variety of diseases.

## Results

### UC-MSCs and fibroblasts shared similar surface markers and morphology

**Fig. 1:**
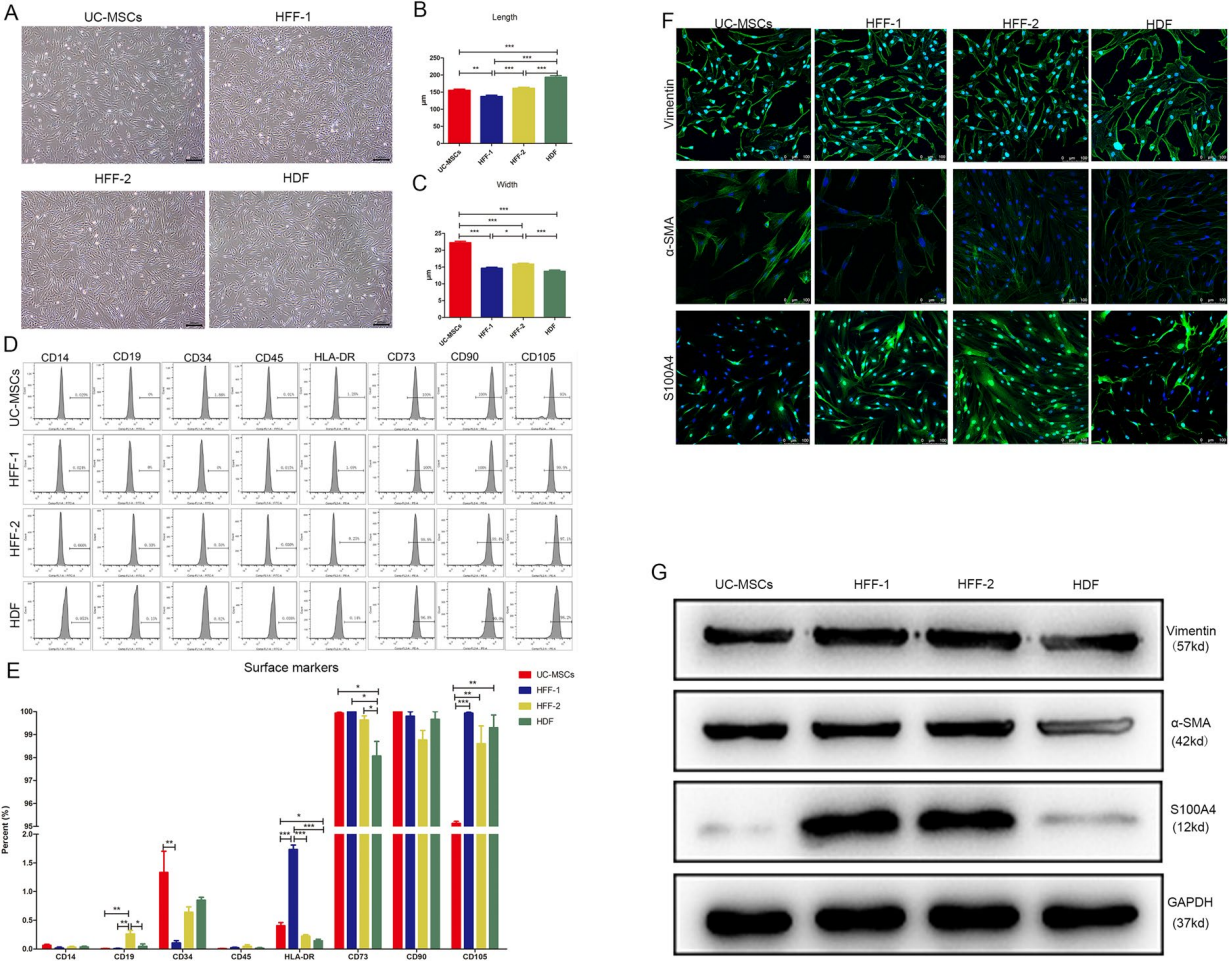
Morphology and surface marker expression profiles of UC-MSCs and fibroblasts. **A** UC-MSCs and three types of fibroblasts at passage 3 shared higly similar long-spindle morphologyunder a microscope. Bar scale = 200 μm. **B**, **C** The quantification of length and width of UC-MSCs and fibroblasts. **D** Flow cytometry was used to detect the surface marker expressions of UC-MSCs and fibroblasts. All types of cells negatively expressed CD14, CD19, CD34, CD45, and HLA-DR, while positively expressed CD73, CD90, and CD105. **E** The quantification of the surface marker expressions of indicated cells. **F**, **G** Expression distribution and level of Vimentin, α-SMA, and S100A4 in UC-MSCs and fibroblasts were tested by immunofluorescence and WB. Bar scale = 100 μm. (*p < 0.05, **p < 0.01, and ***p < 0.001)

To compare the morphology and surface marker expression profile between MSCs and fibroblasts, we chose UC-MSCs, the most widely used type of MSCs, and three types of fibroblasts derived from one eye-widening donor (HDF) and two circumcision donors (HFF1 and HFF2). UC-MSCs and three types of fibroblasts had almost identical morphology, representing long spindle shape under a microscope (Fig. 1A). We further quantified the sizes of four types of cells at passage 3 by Image J software and found some subtle distinctions among them. The lengths of four types of cells were 150 to 200 µm (Fig. 1B). There was obvious heterogeneity in length among HFFs and HDF was the longest cells among them. The UC-MSCs were much wider in cell body than the three types of fibroblasts which shared similar widths (average width: 22 vs. 15 µm) (Fig. 1C). UC-MSCs and three types of fibroblasts shared almost same surface marker expression profile including low expressions of CD14, CD19, CD34, CD45, and HLA-DR, as well as high expressions of CD73, CD90, and CD105. The quantified data showed that there was no significant difference among these eight surface markers except for HLA-DR and CD73 (shown in Fig. 1D, E). Now that fibroblasts shared the classic surface marker expression profile with MSCs, we next investigated whether MSCs also expressed the classic markers of fibroblasts. The α-SMA, vimentin, and S100A4 were reported specifically expressed in fibroblasts. Immunofluorescence and western blot results showed that UC-MSCs indeed highly expressed vimentin and α-SMA, with similarity to three types of fibroblasts, while S100A4 was only highly expressed in HFFs, not in UC-MSCs and HDFs (shown in Fig. 1F, G).

### Fibroblasts had strong multilineage differentiation capacity like UC-MSCs

**Fig. 2:**
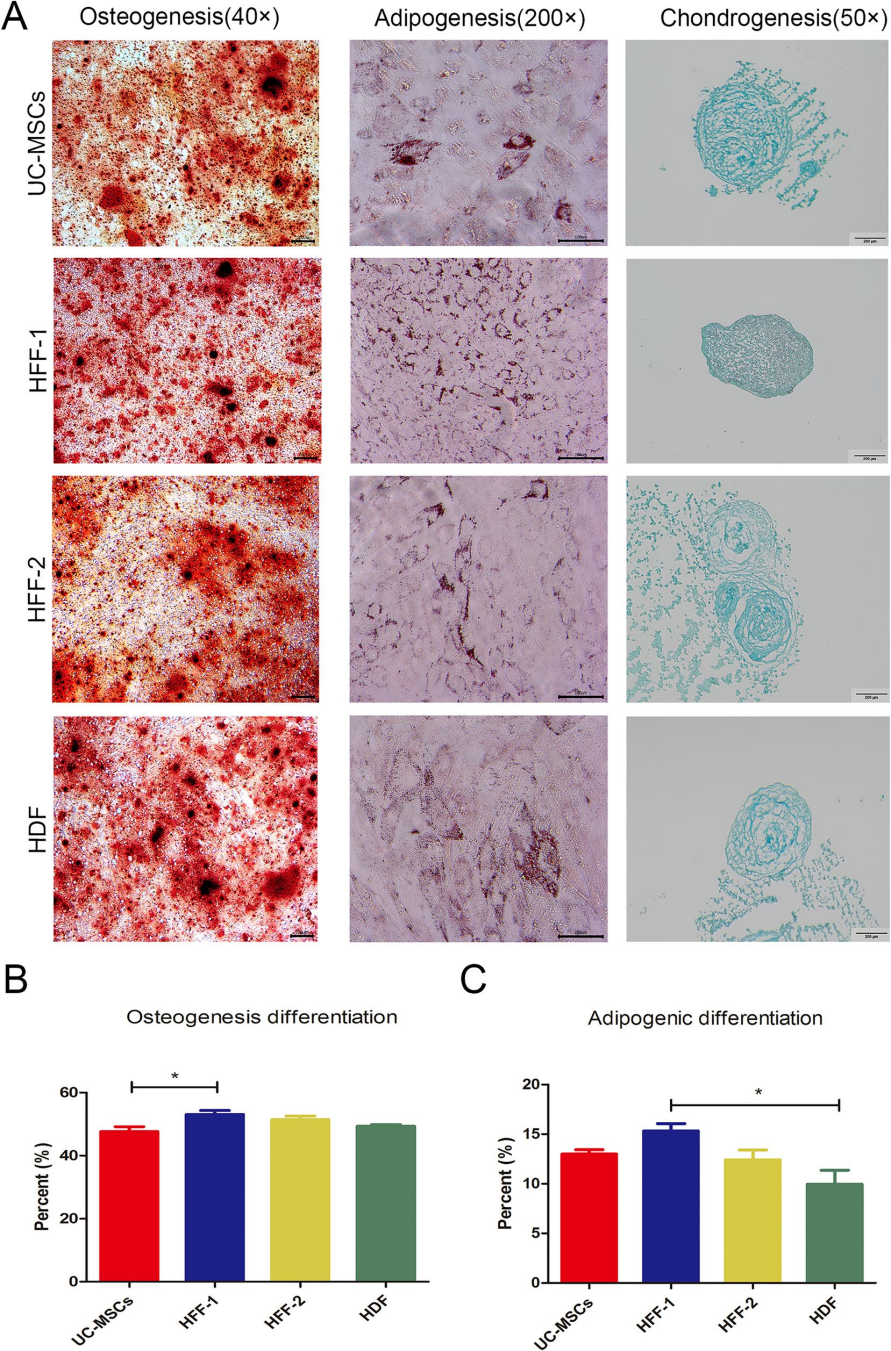
Multilineage differentiation potentials of UC-MSCs and three types of fibroblasts. **A** Osteogenesis, adipogenesis, and chondrogenesis were assayed by Alizarin red-S staining, Oil red O staining, and Alcian blue staining, respectively. **B**, **C** Quantification of osteogenic and adipogenic differentiation. (*p < 0.05, **p < 0.01, ***p < 0.001)

The multilineage differentiation potential is the important feature of MSCs. Over all, fibroblasts had similar osteogenic, adipogenic, and chondrogenic differentiation capabilities to UC-MSCs (Fig. 2A). Quantified data showed that fibroblasts had slight heterogeneity in adipogenic differentiation potential (Fig. 2C), while fibroblasts and UC-MSCs had no heterogeneity in osteogenic differentiation potential (Fig. 2B).

### UC-MSCs and fibroblasts exhibited similar immunomodulatory capacities in vitro

**Fig. 3:**
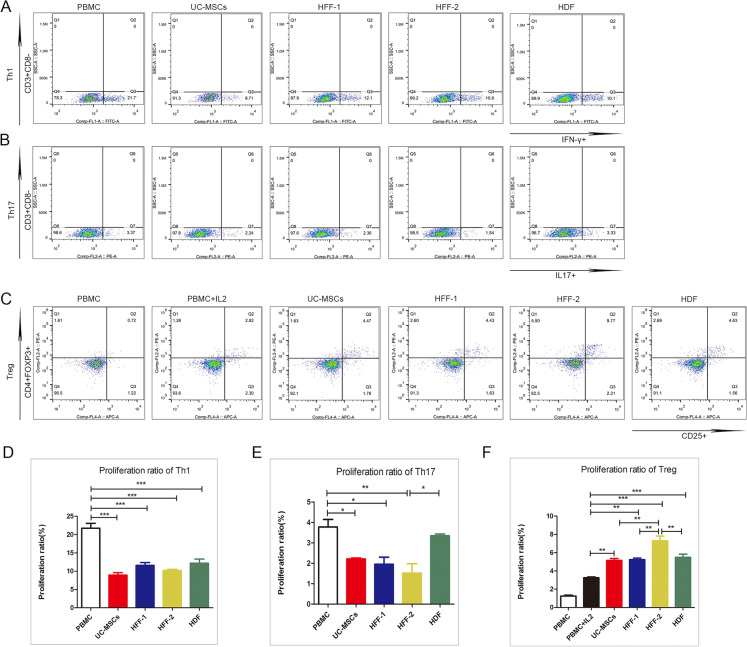
Immunoregulatory capability of fibroblasts and UC-MSCs in vitro. **A**–**C** Flow cytometry was performed to test ratio of Th1, Th17, and Treg. Data were analysed by Flowjo. **D**–**F** Quantification of Th1, Th17, and Treg percentages. (*p < 0.05, **p < 0.01, ***p < 0.001)

Inhibition of Th1/Th17 differentiation and promotion of Treg differentiation are important features of MSCs. We found that all three types of fibroblasts could down-regulate the ratios of Th1 and Th17 and up-regulated the Treg ratio when co-cultured with peripheral blood mononuclear cells (PBMCs), as same as UC-MSCs (Fig. 3A–C). Among three types of fibroblasts, there also were slight heterogeneities in immunoregulation (Fig. 3D–F). Unlike HFFs and UC-MSCs, HDFs did not show evident down-regulation ability of Th17 differentiation (Fig. 3E). UC-MSCs and fibroblasts could both up-regulate the differentiation of Treg cells, compared with control cohort. Among fibroblasts, HFFs-2 had the strongest ability to up-regulate Treg subset, indicating there was evident individual heterogeneity in immunoregulation (shown in Fig. 3F).

### UC-MSCs and HFFs shared expressions of most genes

**Fig. 4:**
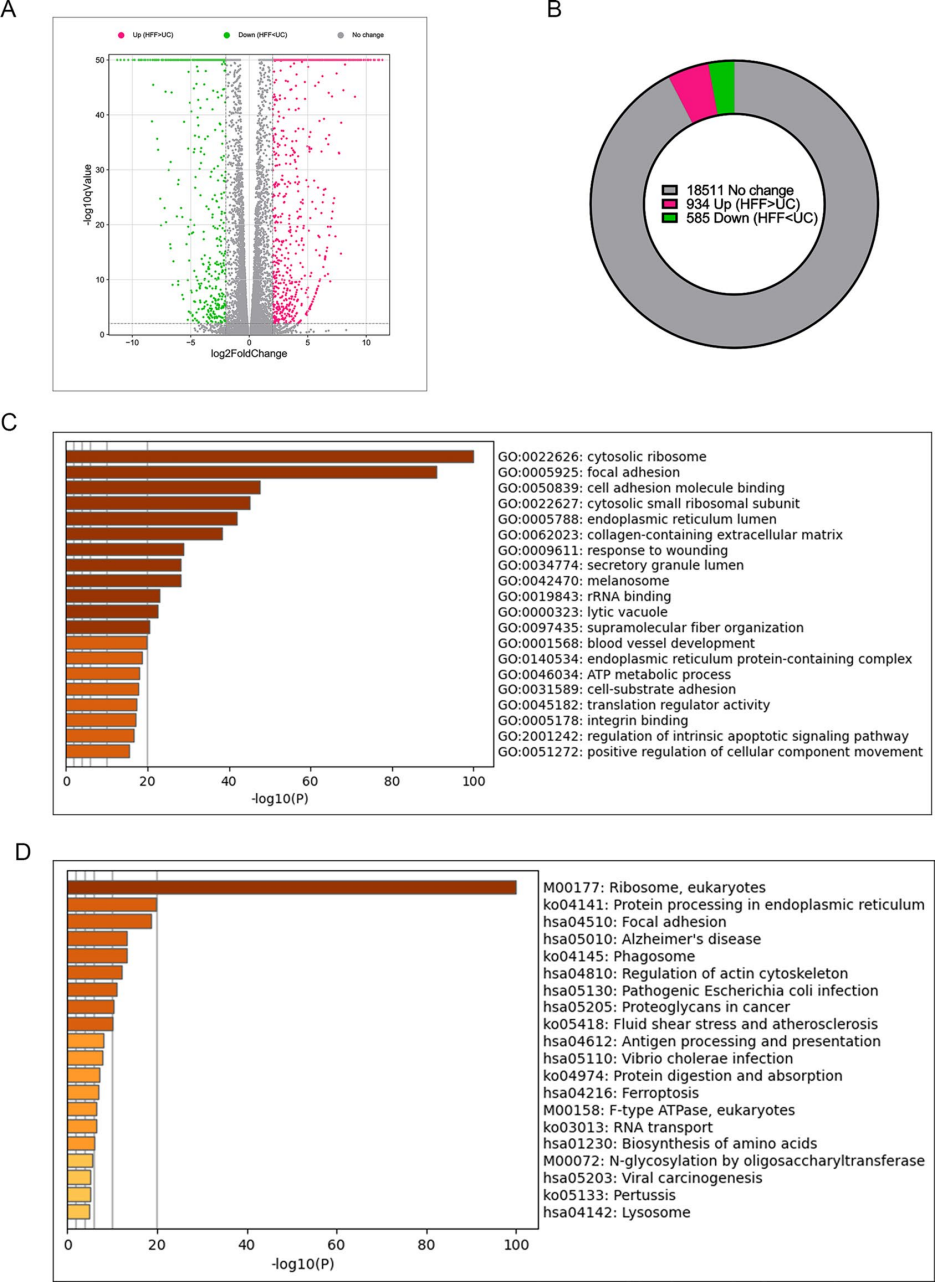

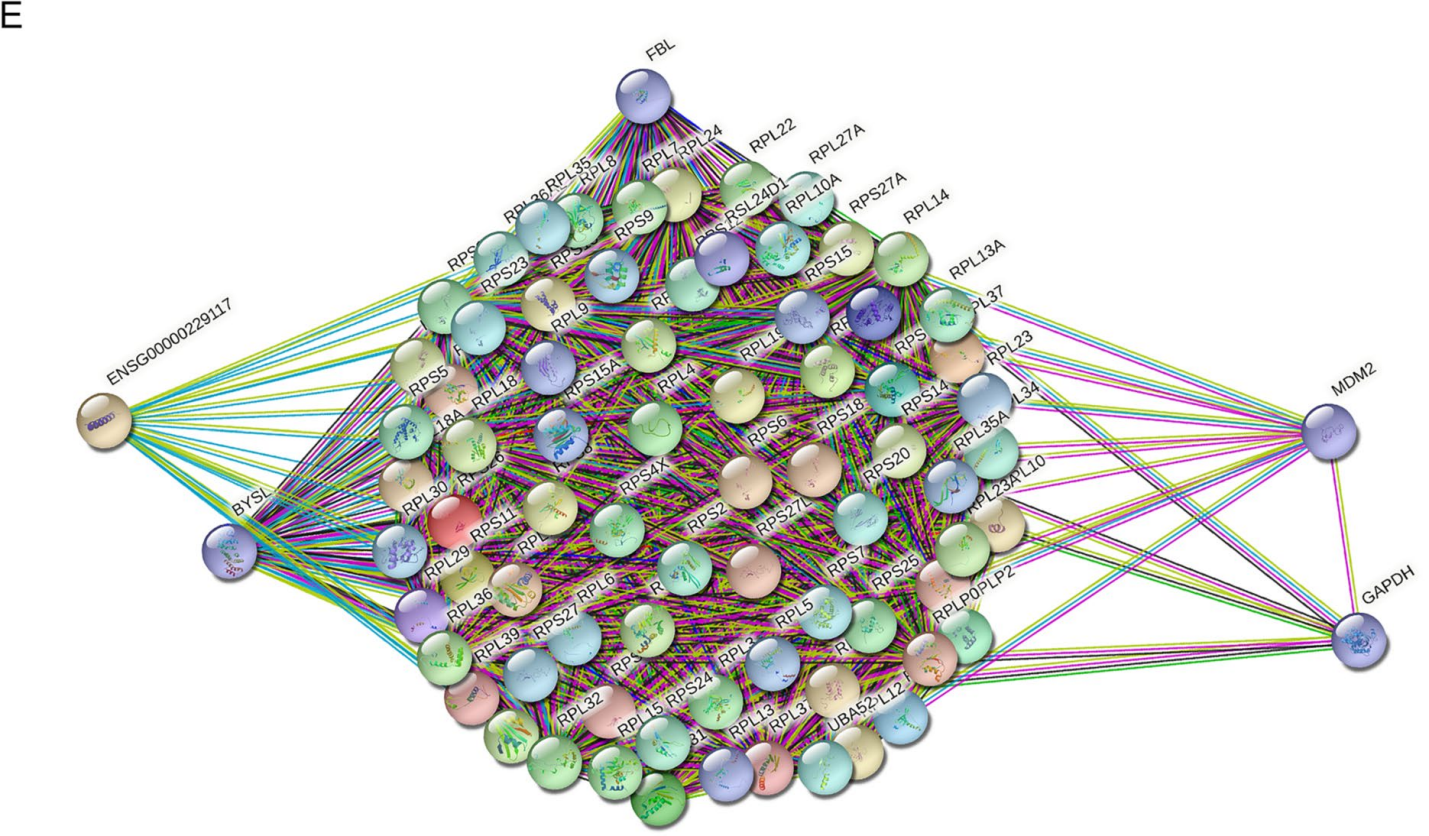
Fibroblasts shared a vast majority of gene expressions with UC-MSCs. **A** Volcano plot showed the basic statistics of differentially expressed genes based on BaseMean from DESeq software. **B** Pie chart showed the proportion of differentially and non-differentially expressed genes based on BaseMean from DESeq software. **C** GO enrichment analysis of top 500 highly co-expressed genes based on FPKM. **D** KEGG enrichment analysis of top 500 highly co-expressed genes based on FPKM. **E** Protein–protein interaction network of 75 highly co-expressed genes in both GO and KEGG ribosome-relative pathways

Based on above experimental results, namely fibroblasts and UC-MSCs had similar biological phenotypes and therapeutic effects on TNBS-induced colitis, we further investigated the similarity of their genic transcriptomes by RNA-seq. HFFs was used as the representative of fibroblasts. Volcano plot showed the basic statistics of differentially expressed genes in Fig. 4A. Pie chart showed the proportion of differentially and non-differentially expressed genes. Compared with 18511 non-differentially expressed genes, there were only 934 up-regulated and 585 down-regulated genes between HFFs and UC-MSCs (|log2FoldChange|> 2, *q-value* < 0.01) (Fig. 4B), indicating that HFFs and UC-MSCs shared similar mRNA expression patterns because the dominant proportion of genes had no significant expression difference (Fig. 4A, B), which were consistent with the functional similarity between two kinds of cell types. Kyoto Encyclopedia of Genes and Genomes (KEGG) pathway and Gene Ontology (GO) enrichment analysis of genes with no difference between the two cells showed strong relations to ribosome composition, which played an important role in protein translation, suggesting that both HFFs and UC-MSCs could produce a large number of active substances (Fig. 4C, D). The pattern of protein–protein interaction (PPI) in HFFs and UC-MSCs showed that the genes related to the ribosome pathway were highly correlated with the expression of FBL, BYSL, MDM2, ENSG, and GAPDH (Fig. 4E).

**Fig. 5:**
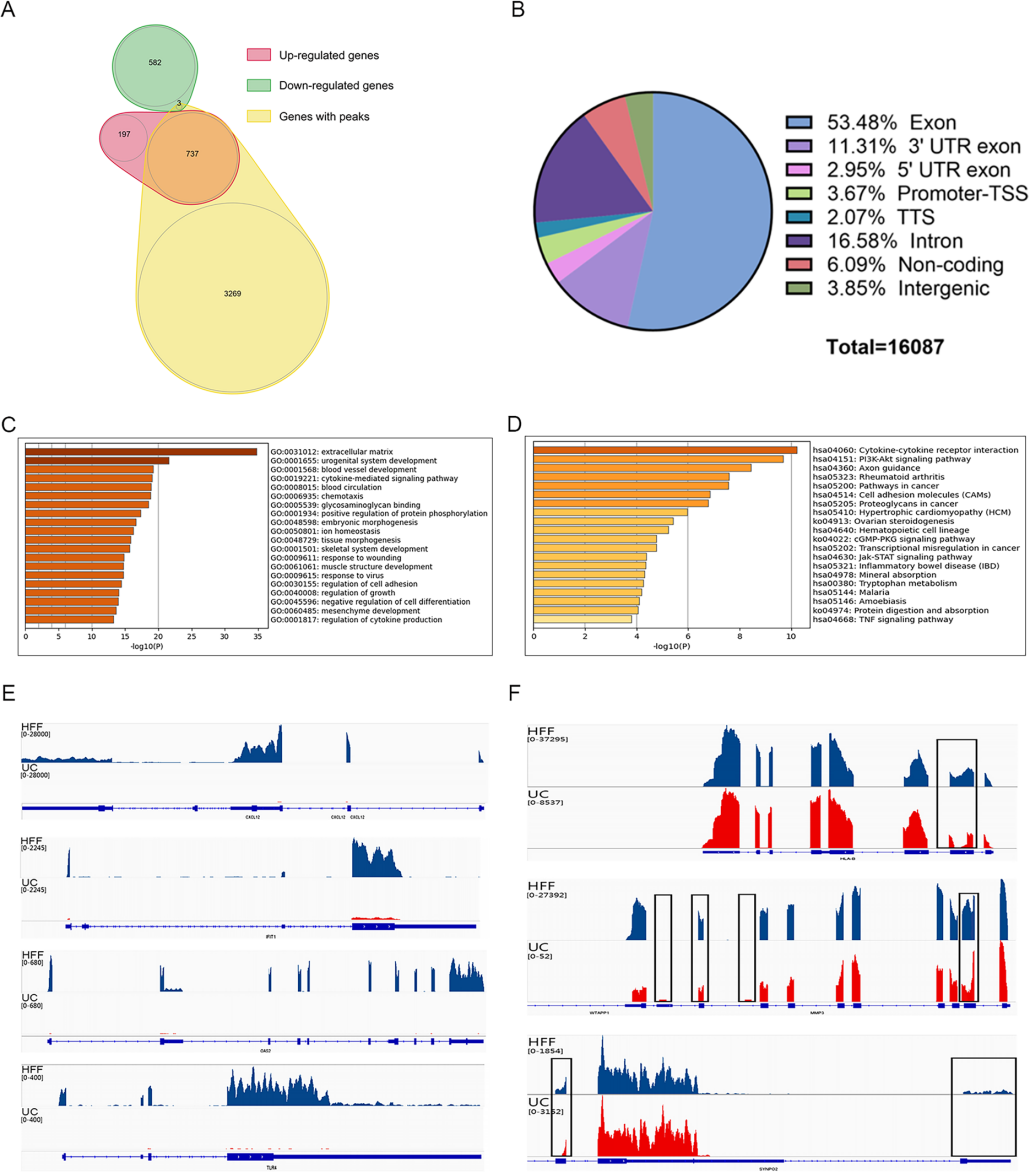
Informatics analysis of differentially expressed genes. **A** Venn diagrams showed relationship of differentially expressed genes using 2 methods, namely DE analysis of genes based on FPKM (red and green) between 2 cell types relative to UC-MSCs and calling of significant HFF-specific expression peaks (yellow) relative to UC-MSCs, as background signal. **B** Genic distribution of significant HFF-specific expression peaks. **C** GO enrichment analysis of 709 HFF-specific expressed genes with significant expression sites (Peaks). **D** KEGG enrichment analysis of 709 HFF-specific expressed genes with significant expression sites (Peaks). **E** Differential genomic sites and differential expression levels were presented between UC-MSCs and HFFs. HFFs’ reads were colored in blue and UC-MSCs’ reads were in red. The range of reads aggregation height were presented on the top left side. **F** Visualized presentation of the HFF-specific peaks, which were highlighted using box, in genes with no change in total expression level between UC-MSCs and HFFs. The meaning of colors and marked information were the same as in Fig. 5E

In order to search for differential pattern of gene expression between HFFs and UC-MSCs, we performed transcriptome sequencing data analysis in two directions. The conventional method was focused on the Differential Expression (DE) analysis based on significance of relative expression value of each gene between three replicates of HFFs and UC-MSCs. At the same time, we used peak-calling tools to get significant clusters of HFFs reads, which was so-called peaks in this article, representing the specific high expression genomic sites of HFFs. For further research, it was important to select genes which differed not only in general relative expression levels, but also at specific genomic sites between two indicated cell types. Venn diagrams showed relationships among groups of differential genes using two methods, including gene sets of DE genes (red and green) between two cell types and genes with significant HFF-specific expression peaks (yellow) relative to UC-MSCs as background (Fig. 5A). There were 737 enrichment sites in up-regulated genes while only 3 enrichment sites in down-regulated genes. Exon accounted for 53.48% of all enrichment sites, which was consistent with the sequencing target of mRNAs together with the consequence of non-mRNA genomic distribution caused by coordinated overlaps of different genes or unknown alternative splicing events (Fig. 5B). However, we also found a small fragment of cell-type specific differentially expressed genes. HFFs highly expressed genes with significant peaks associated with urogenital system and angiogenesis pathways, etc. (Fig. 5C, D). Through the joint analysis of differential genomic sites and conventional differential expression levels, we could obtain information about significantly high expressed transcripts with specific information of expression coordinates of HFFs, such as CXCL12, IFIT1, OAS2, and TLR4 which were significantly higher than those in UC-MSCs (Fig. 5E).On the other way, HFF-specific peaks could be found in genes with no significant expression level change which occupied a dominant position between the RNA expression patterns of the two cell types, such as HLA-B, MMP3 and SYNPO2 (Fig. 5F).

### HFFs and UC-MSCs shared relative similarity of metabolites

**Fig. 6:**
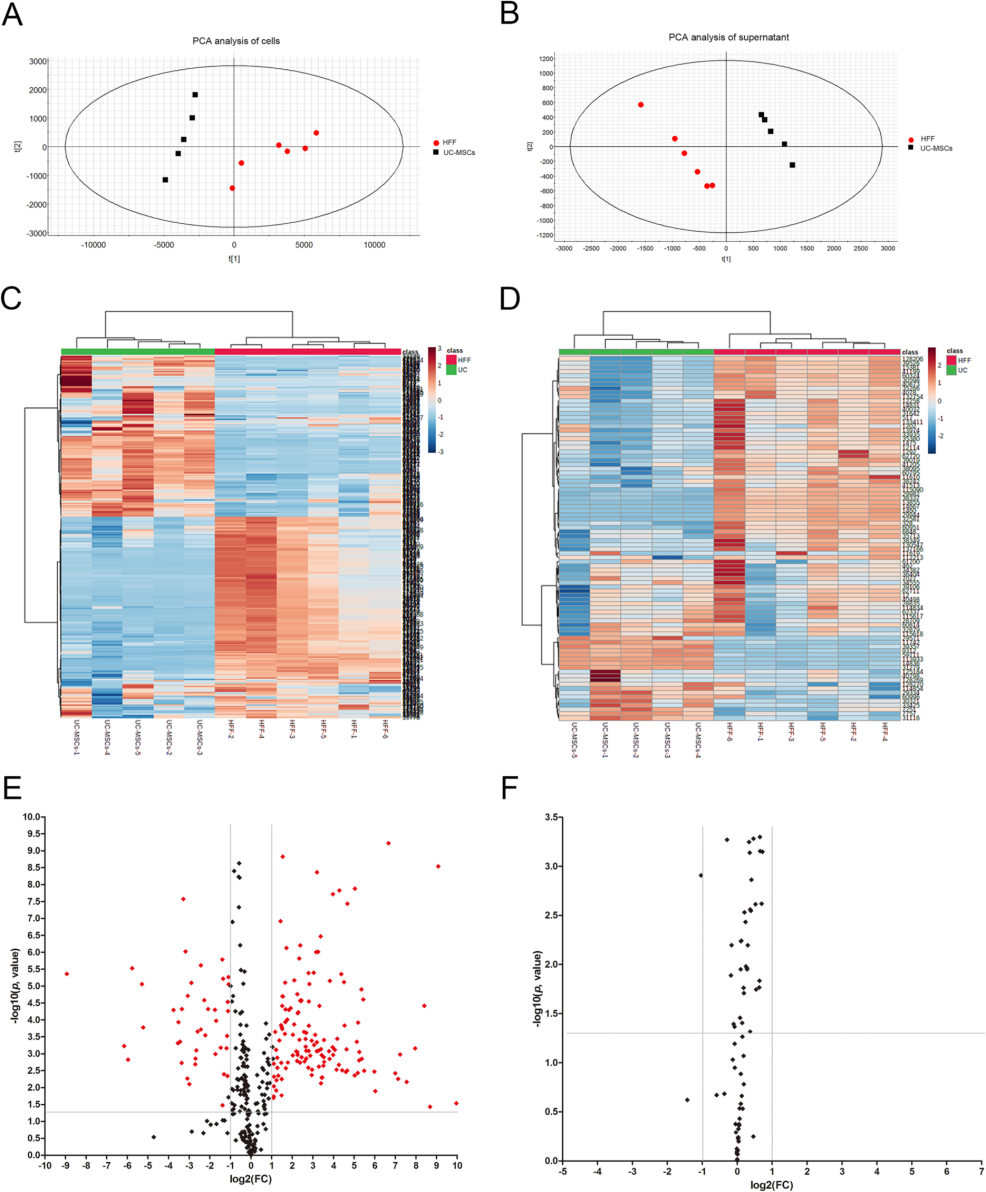

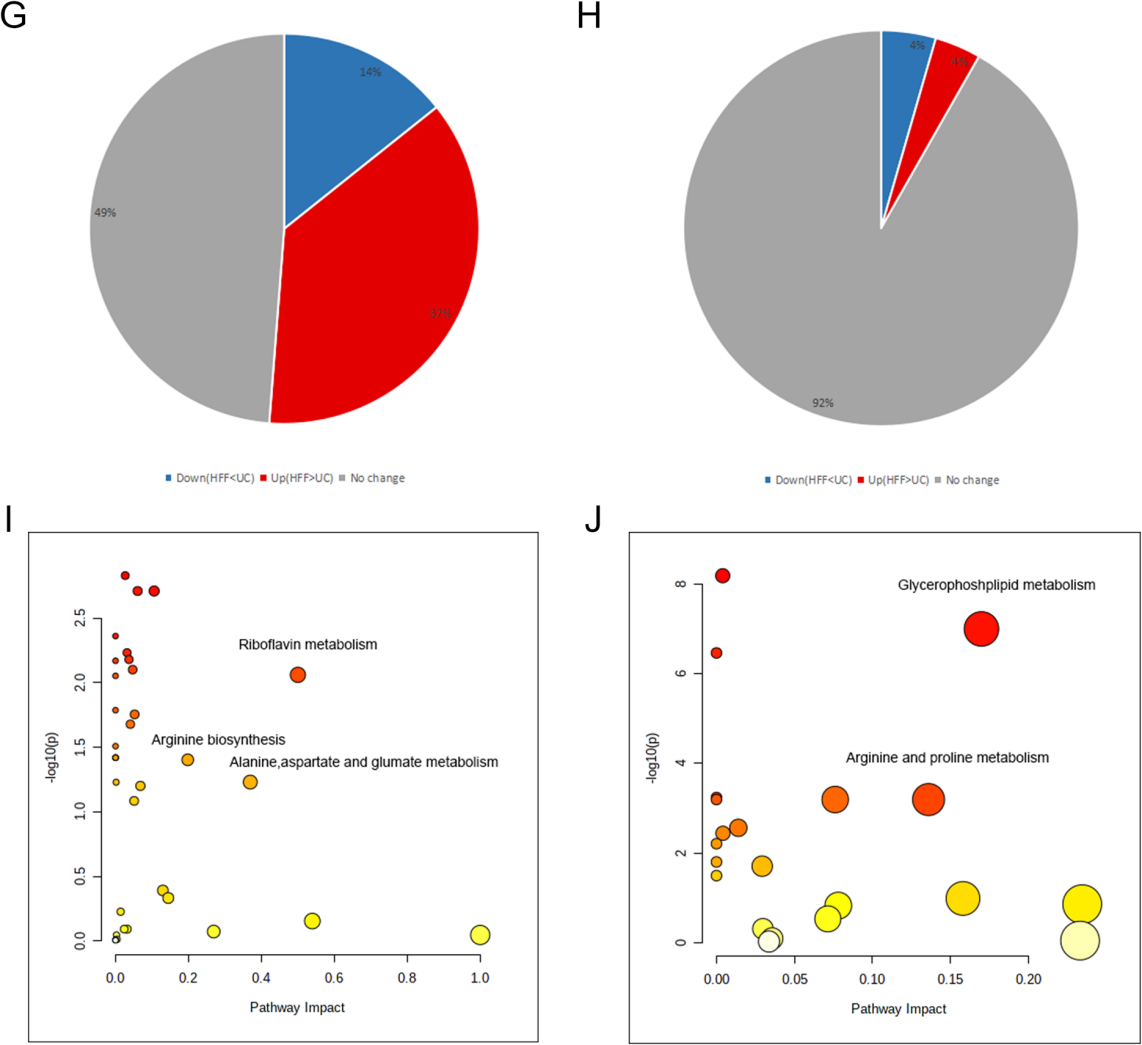
Metabolomic analysis of UC-MSCs and HFFs. **A** PCA analysis of cell lysates. **B** PCA analysis of supernatant. **C** Heat maps of normalized metabolite concentrations in cell lysates. Columns represented the samples, and rows represented the metabolites. **D** Heat maps of normalized metabolite concentrations in supernatant. **E** A volcano plot based on the metabolomic data of UC-MSCs and HFFs. **F** A volcano plot based on the metabolomic data in supernatant. **G** Pie charts of differential metabolites between UC-MSCs and HFFs. **H** Pie charts of differential metabolites in supernatant. **I** Metabolic pathway analysis between UC-MSCs and HFFs. **J** Metabolic pathway analysis of supernatant. (Differential metabolites: *p* < 0.05 and fold change > 2.0 or < 0.5)

Above results demonstrated great similarity of gene expression patterns between UC-MSCs and HFFs, thus we further investigated their similarities and differences of metabolites. The culture supernatants of UC-MSCs and HFFs and their cell lysates were harvested for metabolomic analysis. Principal Component Analysis (PCA) was applied to the metabolite concentration data set (shown in Fig. 6A, B). We could find that PCA clearly separated HFFs from UC-MSCs in both supernatant and cells. Based on the hierarchical cluster analysis of metabolite model, the results of cell lysates and supernatants were presented in the form of heat map (Fig. 6C, D). Volcano diagram (Fig. 6E, F) showed the up-regulated and down-regulated substances of HFFs in supernatant and cell lysates relative to UC-MSCs (*p* < 0.05 and fold change (FC > 2.0 or < 0.5). The pie charts (Fig. 6G, H) showed 92% of substances had no significant difference in supernatants between HFFs and UC-MSCs and nearly half of metabolites showed no difference in cells components between HFFs and UC-MSCs. Pathway analysis of metabolites showed that only a few metabolites showed a significant difference such as riboflavin metabolism, glycerophospholipid metabolism, and arginine and proline metabolism (Fig. 6I, J).

### UC-MSCs and fibroblasts had nearly equal therapeutic efficacy on TNBS-induced colitis

**Fig. 7:**
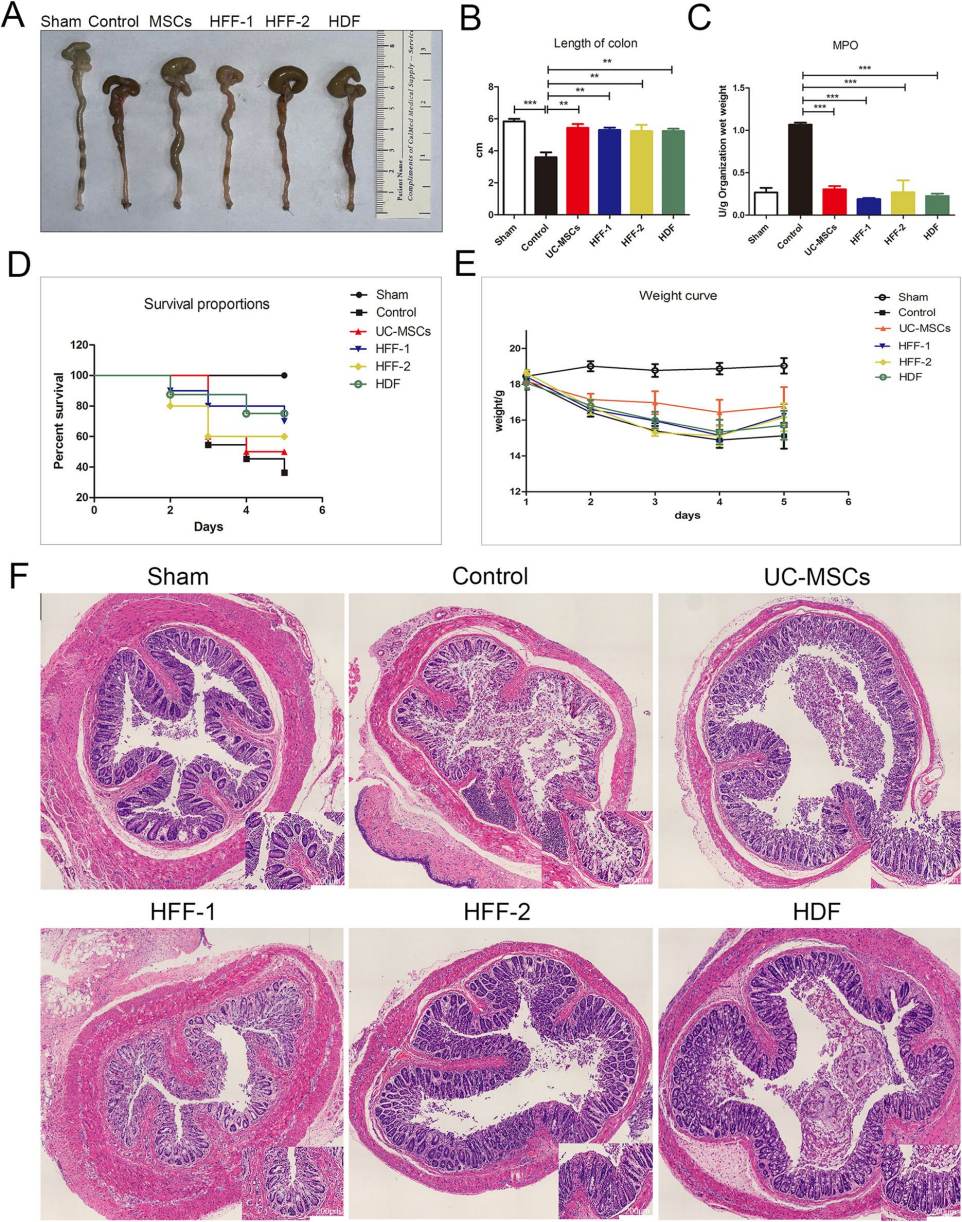
The therapeutic efficacy of fibroblasts and UC-MSCs in treating TNBS-induced colitis. **A** Colon morphology among different groups. **B** The lengths of colons among groups. **C** Quantification of MPO among groups. **D** Survival curve of mice with colitis among groups. (*p* > 0.05). **E** Weight of mice. (*p* > 0.05). **F** H&E staining of the colonic tissues. (**p < 0.01, ***p < 0.001)

Due to similar biological characteristics, especial immunoregulation potential between UC-MSCs and fibroblasts in vitro, we attempted to investigate whether they had similar therapeutic efficacy when applied them to treat TNBS-induced colitis in mice which is a Th cells related disease*.* In TNBS-induced colitis mice, the evident colon hyperemia and shortening colon were observed compared with sham mice, while UC-MSCs and three types of fibroblasts treatments almost equally ameliorated these symptoms (shown in Fig. 7A, B). The neutrophil infiltration was an important feature of colitis model [23]. Myeloperoxidase (MPO) in the model group was significantly increased, indicating the severe neutrophil infiltration in colon. In robust contrast, UC-MSCs and three types of fibroblasts treatments markedly blunted the increase of MPO, without difference among four cell treatment groups (Fig. 7C). The survival and body weight in each group were recorded every day. After five days treatment of cells, the survival rate and weight curve of mice were shown in Fig. 7D, E, with no significant difference among four cell treatment groups (*p* > 0.05). H&E staining of colon tissues showed that compared with the intactintestinal mucosa in the sham group, there was severe damage and many inflammatory cells infiltrated in intestinal mucosa of TNBS-induced colitis mice. The TNBS-induced destructure of colonic mucosa and severe infiltration of immune cells were markedly recovered in all four cell treatment groups (Fig. 7F).

**Fig. 8:**
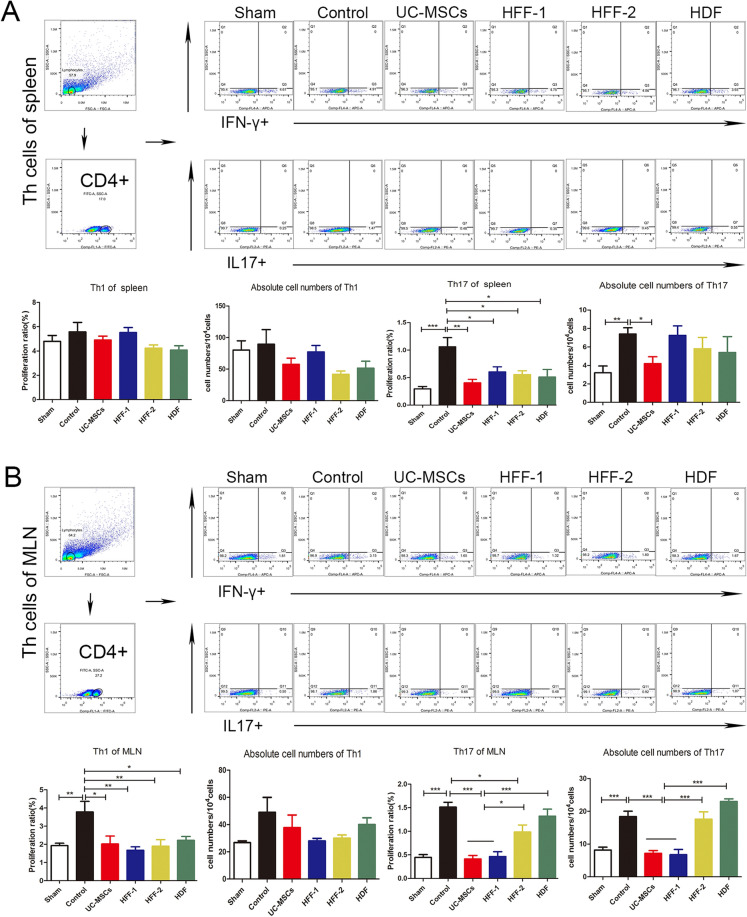

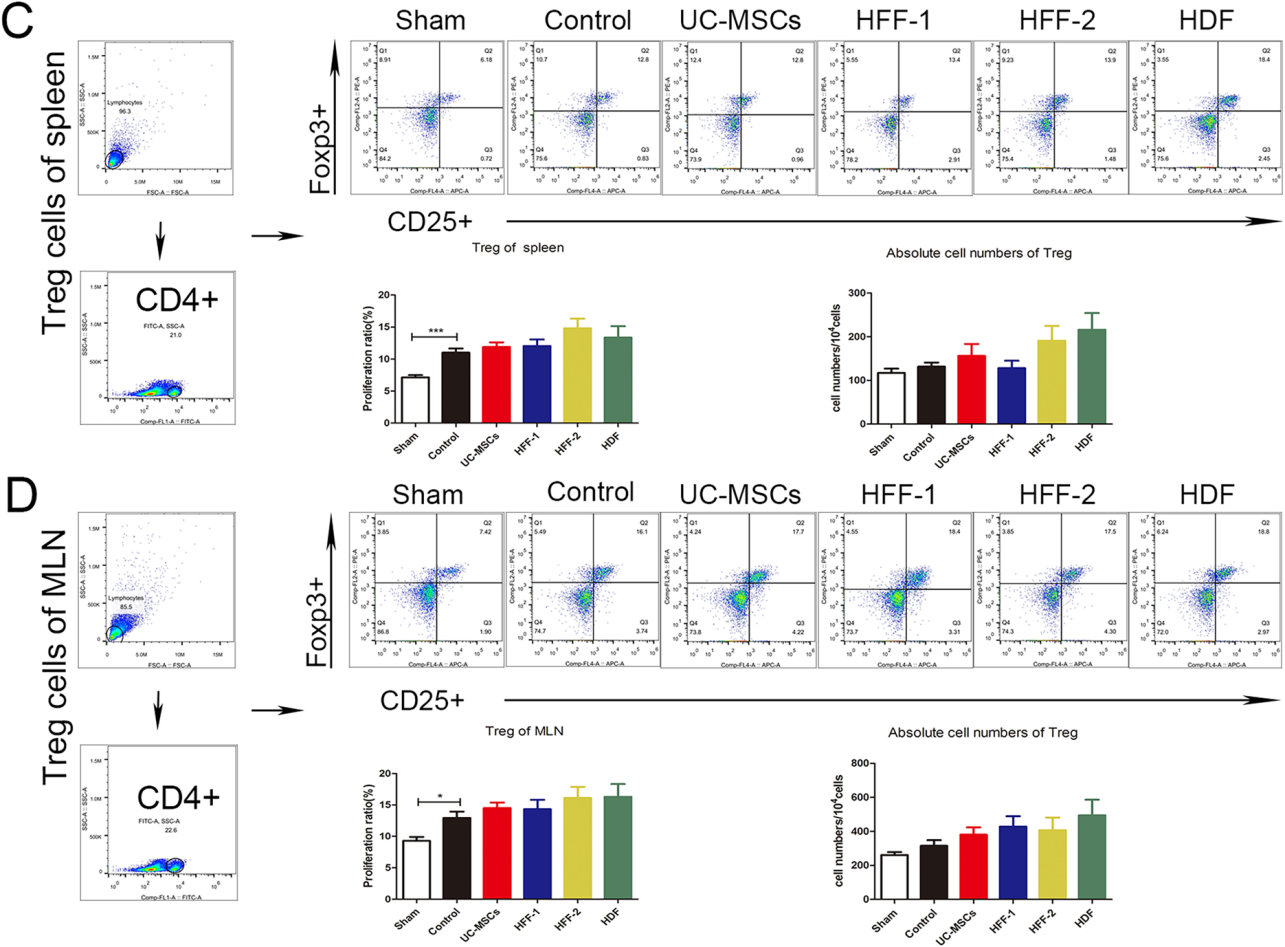
Immunoregulatory effects of fibroblasts and UC-MSCs in vivo*.* **A** Immunoregulation of Th1, Th17 in spleens. **B** Immunoregulation of Th1, Th17 in MLNs. **C** Immunoregulation of Treg in spleens. **D** Immunoregulation of Treg in MLNs. (*p < 0.05, **p < 0.01, ***p < 0.001)

The cell suspensions of spleens, mesenteric lymph nodes (MLNs) and lamina propria (LP) were prepared for investigating the effects of UC-MSCs and fibroblasts on immune regulation in vivo. Altogether, three types of fibroblasts showed similar in vivo immune regulation potentials with UC-MSCs including decreasing percentage of Th1 and Th17, and increasing percentage of Treg cells in spleens and MLNs (Fig. 8A–D), as well as that could be seen in the LP (Additional file 1: Fig. S1A, B). In UC-MSCs and HFF-1 treatment groups, neutrophils had a significant decreasing trend in LP (Additional file 1: Fig. S1C) while M1-type macrophages decreased significantly in UC–MSCs and HFF-2 treatment groups (Additional file 1: Fig. S1D).

### UC-MSCs and HFFs had similar therapeutic efficacies on hepatic fibrosis

**Fig. 9:**
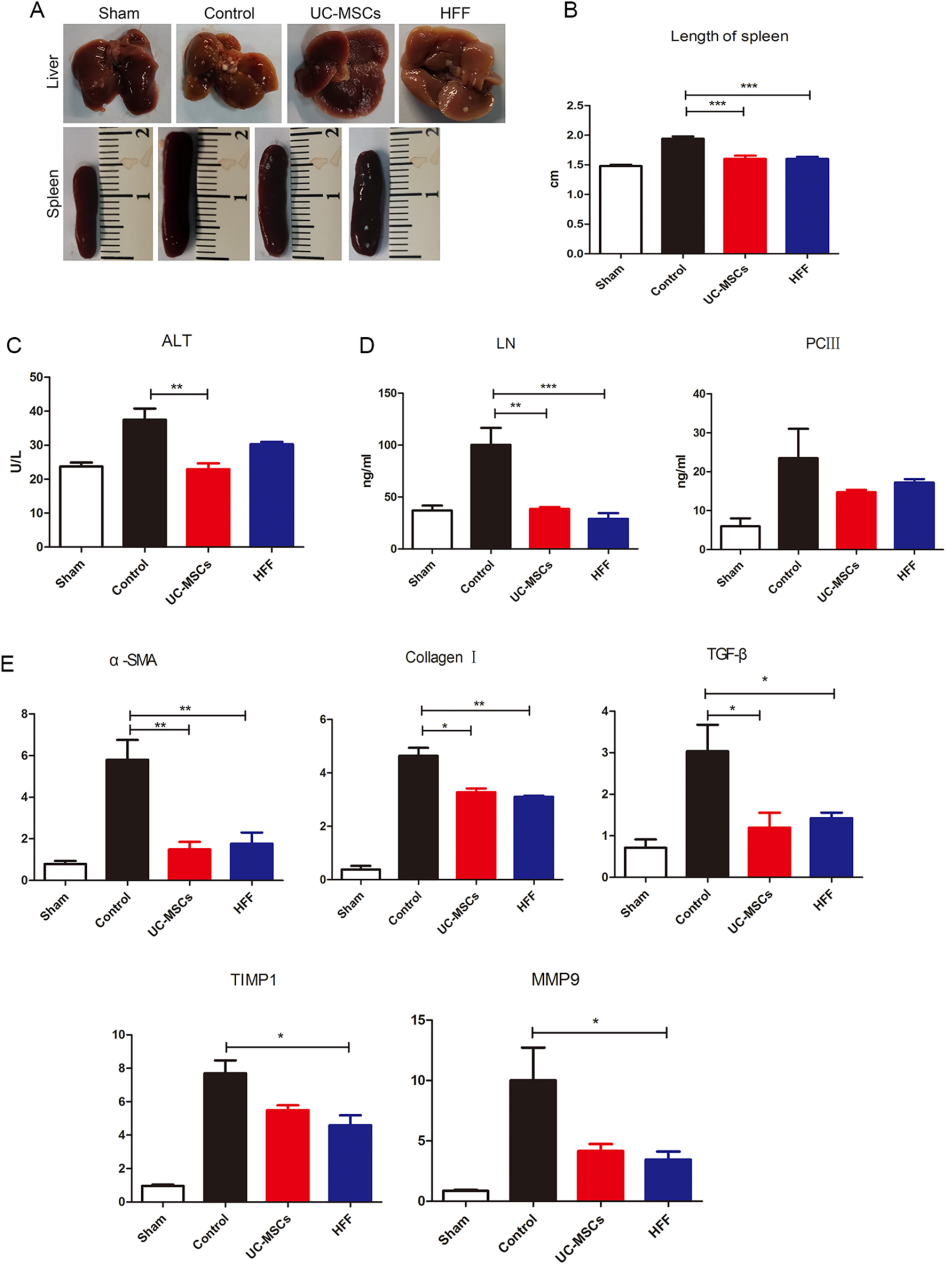
The therapeutic efficacy of UC-MSCs and HFFs applied to hepatic fibrosis. **A** Liver and spleen morphology among groups. **B** Statistical analysis of the length of spleens. **C** ALT levels in serum among groups. **D** LN and PCIII levels in livers among groups. **E** The indicators of livers fibrosis were detected by Q-PCR. (*p < 0.05, **p < 0.01, ***p < 0.001)

**Fig. 10:**
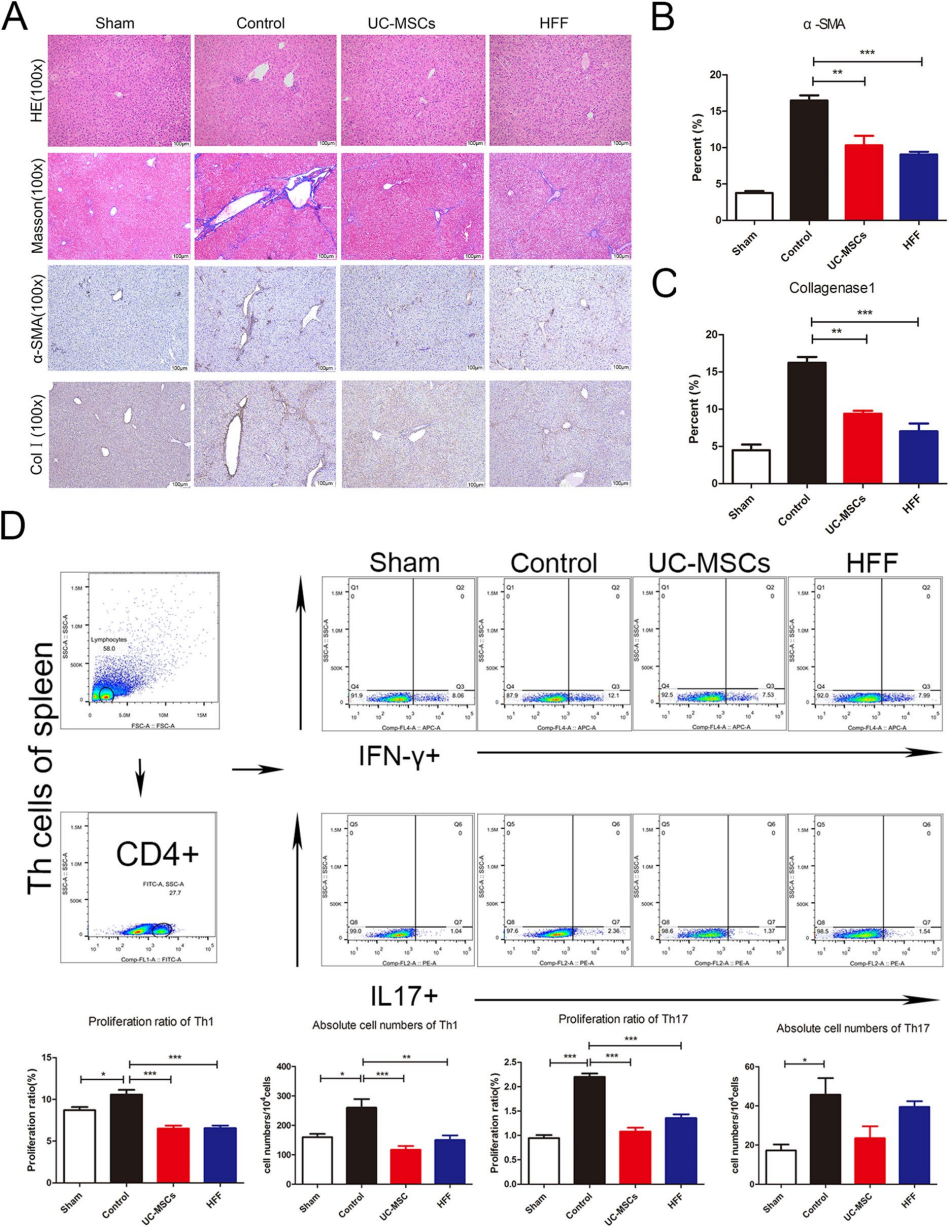

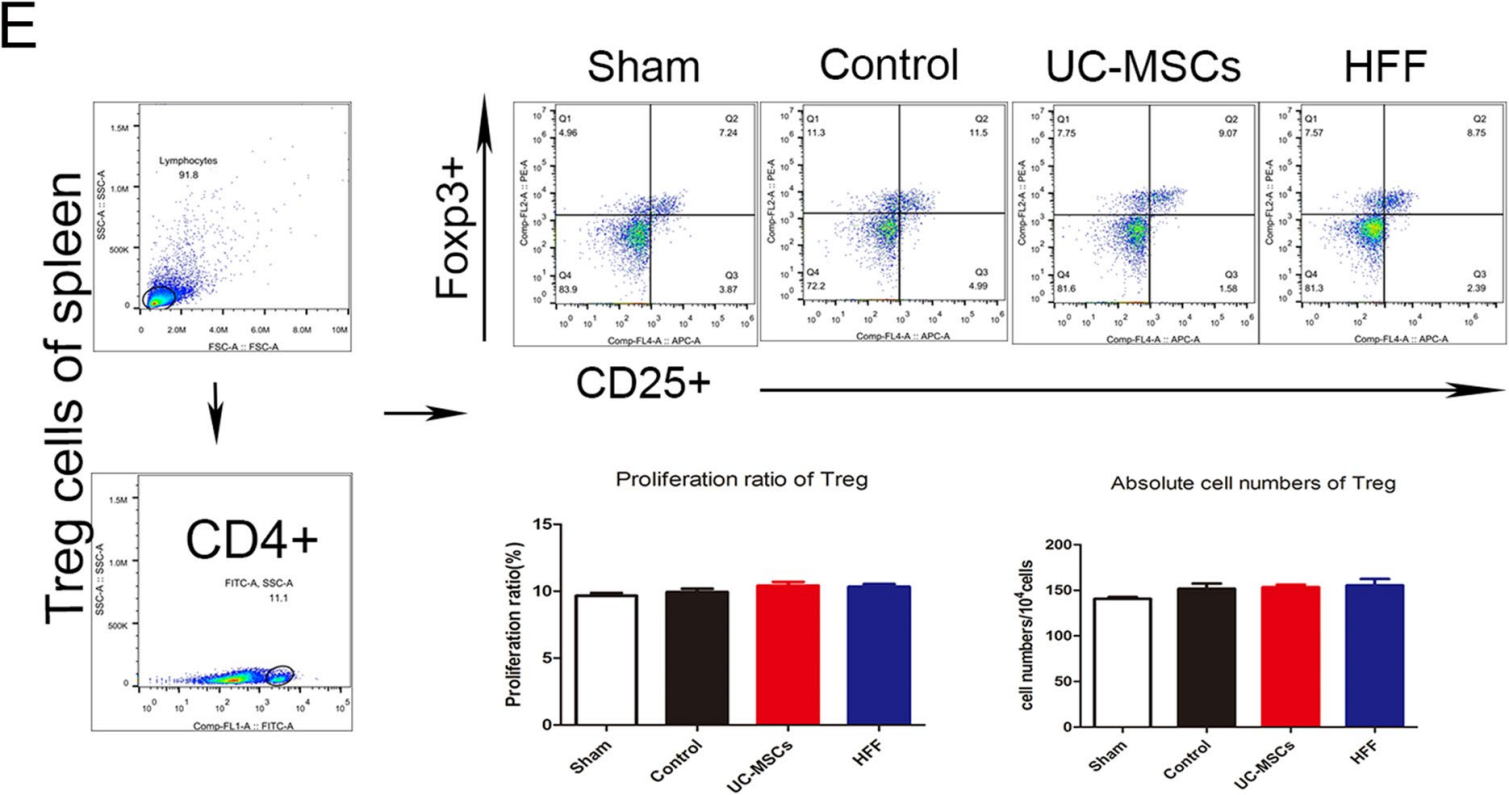
Fibroblasts and UC-MSCs markedly alleviated the liver fibrosis. **A** H&E staining, Masson staining, and immunohistochemistry against α-SMA and collagen I. **B**, **C** Quantification of α-SMA and collagen I immunostainings. **D** Immunoregulation potentials of Th1 and Th17 in spleens. **E** Immunoregulation potentials of Treg in spleens. (*p<0.05, **p < 0.01, and ***p < 0.001)

Since UC-MSCs and HFFs have been shown largely similar in the gene expression and metabolites indicating that fibroblasts and UC-MSCs indeed had great similar or equal biological features, contributing to corresponding similar therapeutic efficacy in disease treatment. We further validated their therapeutic efficacies when applied to another disease model, hepatic fibrosis which was widely treated by various kinds of MSCs. Hepatic fibrosis nodules and enlargements of spleens were evidently induced by CCL_4_ in model mice, however both HFFs and UC-MSCs treatments could markedly blunt the CCL_4_-induced hepatic fibrosis nodules and enlarged spleens (Fig. 9A, B). ALT level indicated the degree of liver injury and CCL_4_ treatment significantly increased the serum ALT levels in mice, which were significantly alleviated by HFFs and UC-MSCs treatments (Fig. 9C). HFFs treatment also could down-regulate the LN and PCIII of liver tissues, the indicators of liver fibrosis evaluated by ELISA (Fig. 9D) and significantly decreased α-SMA, collagen I, TGF-β, TIMP1, and MMP9 expressions evaluated by Q-PCR, similar to UC-MSCs treatment (Fig. 9E). Both HFFs and UC-MSCs treatments markedly alleviated the CCL_4_-induced infiltrations of inflammatory cells in liver assayed by H&E staining, aggregation of collagen assayed by Masson staining, positive rates of α-SMA and collagen I assayed by immunohistochemistry (Fig. 10A–C). In addition, the ratios of Th1 and Th17 in the spleens were markedly decreased after HFFs and UC-MSCs treatments (Fig. 10D, E). These results clearly indicated that HFFs and UC-MSCs had similar therapeutic efficacies in liver fibrosis to a large extent.

## Discussion

The purpose of this study was to compare the differences and similarities between UC-MSCs and various types of fibroblasts in terms of surface markers, multilineage differentiation, immune regulation, mRNA expression profile, metabolomics analysis, and therapeutic efficacy in treating diseases. The results showed that various types of fibroblasts and UC-MSCs shared similar surface markers and were slightly varied in morphology of long spindle shape. All three types of fibroblasts had strong multilineage differentiation capacity like UC-MSCs including osteogenic, adipogenic, and chondrogenic differentiation capabilities. UC-MSCs and fibroblasts exhibited similar abilities to inhibit Th1/Th17 differentiation and promote Treg differentiation in vitro. UC-MSCs and HFFs shared great similarities in mRNA expression profile and metabolites, contributing to their similar biological characteristics. In addition, UC-MSCs and fibroblasts had nearly equal therapeutic efficacy on TNBS-induced colitis and CCL_4_-induced hepatic fibrosis.

Surface marker expression profile is often used to define a certain kind of stem cells. It is difficult to distinct MSCs from fibroblasts according to their surface marker expressions because the general surface markers used to define MSCs are also found in fibroblasts. In present study, we found fibroblasts had almost same classic surface marker expression profiles which were used to define MSCs such as low expressions of CD14, CD19, CD34, CD45, and HLA-DR, as well as high expressions of CD73, CD90, and CD105. In addition, some classic surface markers such as Vimentin and α-SMA which were considered to be expressed fibroblasts also were highly expressed in UC-MSCs. Our study again proved that it was available to precisely define MSCs or fibroblast using a set of surface marker expressions. Notably we found that although fibroblast and UC-MSCs had almost identical long-spindle morphology, UC-MSCs were much wider than three tested types fibroblasts (width: 22.50 μM vs. 14.80 μM). Thus, we proposed that we could distinct fibroblasts from MSCs according to the width of their cell body. In addition, we also noticed that there was evident tissue heterogeneity in surface marker expression of fibroblasts. Currently, S100A4 is generally used to identify fibroblasts, but S100A4 was only highly expressed in HFFs comparing to HDFs in our study. S100 protein interacts intracellularly with enzymes, cytoskeletal subunits, receptors, transcription factors, and nucleic acids. They participate in the regulation of proliferation, differentiation, apoptosis, Ca^2+^ homeostasis, energy metabolism, and inflammation migration. Some S100 proteins activate surface receptors (such as late glycosylated end-product receptors and toll-like receptor 4), G-protein-coupled receptors, heparin sulfate proteoglycans, and n-glycans, regulating cellular function through autocrine and paracrine. S100A4 and S100B also interact with epidermal growth factor and basic fibroblast growth factor (bFGF) respectively to enhance the activity of corresponding receptors [24]. It has been reported that HFFs presenting high expression of S100A4 may be related to the biological function [25]. Thus, specifically expressed S100A4 may also have specific biological function different from other types of fibroblasts.

Besides similar surface marker expression profile with MSCs, the dermal fibroblasts had same multilineage differentiation potential and immunoregulation capability as UC-MSCs. Our study showed that both UC-MSCs and fibroblasts presented potent osteogenic, adipogenic, and chondrogenic differentiation potentials with no significant differences. Previous studies have reported that UC-MSCs and fibroblasts came from mesoderm, which proposed the hypothesis that fibroblasts may be aged MSCs [26]. We suspected that UC-MSCs and fibroblasts owned the similar differentiation capability due to their homology such as common origination from embryonic stem cells. When we co-cultured UC-MSCs and fibroblasts in vitro with human PBMCs respectively, all these cells demonstrated evident immunoregulatory ability to regulate differentiation of T lymphocytes mentioned in Mehdi Najar’s study [27, 28]. T lymphocytes are the key adaptive immune regulatory cells involved in fibrosis and inflammation [29–32].

Because we found three types of dermal fibroblasts all had similar biological features, we further explored the deep intrinsic relationship between MSCs and fibroblasts (HFFs as representative fibroblasts) by transcriptome sequencing and metabolomics. The transcriptome sequencing showed that HFFs shared the vast majority of genes with UC-MSCs and only very small fragment of genes were differentially expressed between UC-MSCs and HFFs. GO and KEGG pathway analysis indicated that non-differentially expressed genes were highly related to the ribosome formation pathway. They were also put into analysis for protein–protein interaction and results showed their close functional interactions with FBL, BYSL, MDM2, ENSG, and GAPDH proteins. These proteins are involved in embryonic implantation and development except for GAPDH, supporting the hierarchy homology of UC-MSCs and HFFs. UC-MSCs and HFFs potentially differentiated from embryonic stem cells may explain their absence of significant differences in phenotype and functions in vitro and in vivo. Interestingly, GO and KEGG enrichment analysis of differentially expressed genes between UC-MSCs and HFFs revealed that up-regulated genes relating to HFFs were associated with genitourinary system, blood circulation, and prevention of viral infection, the difference perhaps confirmed the tissue specificity of cells isolated from different tissues [33]. We performed a differentially expressed locus analysis of up-regulated genes in HFFs by using statistical tools looking for genomic information on reads with significantly enriched in HFFs relative to UC-MSCs, which was the genomic information of the loci of peaks. Through combining analysis of differential expression sites together with conventional differential expression levels, we could obtain information about significantly highly expressed transcripts or even binding sites of HFFs, such as CXCL12, IFIT1, OAS2, TLR4 which were significantly higher than those in UC-MSCs. IFIT1 and OAS2 were closely related to the activation of the autoimmune system due to virus infection. Their high expressions may be related to the isolation sites of fibroblasts. Giri et al. found that MSCs could up-regulate the expression of IL-10 in CCR2 + macrophages by secreting CCL2 and CXCL12, enabling cytokine to reach the desired disease site and play a role [34]. TLR4 is a Toll-like receptor and has been reported to have a repairing effect on DSS-induced intestinal injury and up-regulation of IL6, CCL2 and CSF3 [35], which may also be related to their therapeutic effects on TNBs-induced colitis. Metabolomic analysis displayed that there was a little of difference between UC-MSCs and fibroblasts in the culture supernatant, which may be related to the complex components in the culture medium. The cell lysates were also collected for metabolomic analysis, although PCA and heat maps can distinguish UC-MSCs and HFFs, there were still nearly half of the substances which did not meet our screening criteria for differential substances. Therefore, together with the sequencing results, we could speculate that this was due to the similarity of mRNA expressions and metabolites between UC-MSCs and fibroblasts, which may lead to their similar biological functions. Of course, they did have differences between the two kinds of cells, which need to explore their specific biological functions in the future.

We used the acute colitis model to verify the immune regulatory of UC-MSCs and three types of fibroblasts in vivo. UC-MSCs and fibroblasts had functionally equivalent therapeutic efficacy on colitis. In addition, UC-MSCs and three types of fibroblasts similarly regulated the T lymphocyte differentiation in spleens and especially in MLNs. MLNs lymphocytes were more sensitive in the response to sites of inflammation due to their local immune capacity [36]. We also evaluated the changes of CD4 + T cells, macrophages, and neutrophils in intestine.

We selected UC-MSCs and HFF-1 to re-test their ability to treat diseases due to their encouraging efficacy on colitis. Previous studies suggested that HFFs could propel fibrosis [37], but our study verified that HFFs had the same therapeutic capacity in CCL_4_-induced liver fibrosis as UC-MSCs. ALT, PCIII and LN are indicator of liver damage. The high expression of α-SMA is a hallmark of hepatic hepatastrocytes (HSCs) activation [38]. TGF-β is a class of multifunctional cytokines that plays an important role in the development of liver fibrosis, which can promote the synthesis and secretion of extracellular matrix, and reduce the degradation of extracellular matrix. High expression of TGF-β can promote tissue fibrosis and related to the severity of pathology. Pathologic diseases resulted from collagen excessive deposition in livers due to synthesis and degradation of disorder in extracellular matrix. Matrix metalloproteinases (MMP) and metalloproteinases tissue inhibiting factor (TIMP) are the main enzymes regulating the degradation of extracellular matrix. MMP promotes the degradation of extracellular matrix while TIMP prevents the degradation of extracellular matrix by inhibiting MMP. The increase of TIMP levels contributes to disease development [39]. TIMP1 and MMP9 were both raised in the liver fibrosis. HFFs treatment could equivalently alleviate above CCL_4_-induced indicators of liver fibrosis, like the UC-MSCs treatment. Additionally, HFFs also had the same immunoregulatory effects on T lymphocytes differentiation in spleen as UC-MSCs.

Dermal fibroblasts are the main resident cells in dermis, which produce the ECM and contribute to the initiation and circulation of hair follicles. It has been reported that dermal cells derived from different embryonic sites. Ventral, flank trunk, and limb dermis originated from lateral plate mesoderm while craniofacial dermis in mammals has dual embryonic origins [40]. The similarity of fibroblasts and MSCs in mesoderm origin may lead to a certain functional correlation. Ryan R et al. [14] also confirmed that dermal fibroblasts were formed by different lineages and had a remarkable diversity of functions. Some studies based on the definition of MSCs and fibroblasts had not found any characteristics that could distinguish MSCs from fibroblasts [41, 42]. In present study, we isolated the skin dermis-derived fibroblasts according to the classic isolation and culture protocol and obtained the cells with universal nomenclature of “fibroblasts”. We only detect minor differences between fibroblasts and MSCs. Compared with UC-MSCs, the cell body width of three tested dermis-derived fibroblasts was smaller and the reported fibroblast marker, S100A4 were specifically positively expressed in fibroblasts derived from human foreskins (HFFs). Until now, with our knowledge, there is no well-established method to distinguish dermal fibroblasts from MSCs. In current study, we showed that human skin dermis-derived fibroblasts in the universal nomenclature were a kind of functional MSCs-like cells based on the judgements from surface markers, biological characteristics, to therapeutic efficacy. Thus the skin dermis-derived fibroblasts potentially comprise sub-population of skin-derived MSCs or even are equal to skin-derived MSCs, which is worth further being investigated using single cell sequencing technology in further study.

In all, our study suggested that human skin dermis-derived fibroblasts were a kind of potent immunoregulatory and functional mesenchymal stromal cells in treatment of some disorders. In the perspective of cell-based therapy, fibroblasts provide accessible and alternative sources.

## Conclusion

In conclusion, we comprehensively investigated the similarities and differences between MSCs and fibroblasts including phenotypes, multilineage differentiation, and immunoregulatory capacity in vitro and therapeutic efficacy in vivo. The results showed that human skin dermis-derived fibroblasts were a kind of functional MSCs and had equivalent therapeutic efficacy of MSCs in treating specific complications. Our study provided us further understanding of MSCs and fibroblasts and indicated that fibroblasts had a remarkable potential as alternative cells for MSCs in treatments of a variety of diseases.

## Materials and methods

### Cell isolation and culture

Human umbilical cord tissues were obtained from healthy donors after delivery and handled within 1 h. Dulbecco's Phosphate Buffered Saline (DPBS, Gibco, A12858-01, NY, USA) containing 1% Penicillin–Streptomycin (PS, Gibco, 15140122, NY, USA) was used to wash tissues three times and umbilical cord was cut into fragments with sterile surgical instruments. A total of 10 ml type I collagenase (Gibco, 17100017, NY, USA) including 2.5 U/mL Dispase II (SIGMA, D4693, NY, USA) was used to digest tissues in shaking bed (37 °C, 220 r/min). The digestion process lasted for 2 h and was terminated by adding complete medium (DMEM, Gibco, 10567014, NY, USA) containing 10% fetal bovine serum (FBS, Gibco, 12664025, NY, USA). Cell pellets were inoculated in culture flask with complete medium and cultured in a cell incubator (37 °C, 5% CO_2_) after centrifugation at 300 g for 15 min. The nonadherent cells were discarded by changing complete medium after 48 h. MSCs growing up to 80–90% confluence were defined as the primary passage (P0).

Foreskin tissues were obtained from the departments of andrology, Nanjing Drum Tower Hospital. Foreskin from a healthy male after circumcision was taken to the laboratory with 0.9% normal saline and was rinsed 3 times in PBS containing 2% penicillin–streptomycin. Subcutaneous tissue was removed as clean as possible with sterilized ophthalmic scissors and tweezers. The digested foreskin was taken out and the epidermis and dermis were carefully separated after expanding the processed foreskin and putting it into a 37°Cincubator for digestion for 2 h within 0.5% dispase. The dermis was digested with 0.2% type I collagenase for 2 h in an incubator at 37°C then the digestion was terminated with complete medium and the dermal tissue digested was taken out. The fibroblasts were collected into a 50 ml centrifuge tube by twice supernate filtration using 200 mesh sterilization filters followed with a centrifugation at 1500 rpm for 5 min. The cells were re-suspended with the complete culture medium (DMEM containing 10% FBS), then placed in 25cm^2^ flasks (5%CO2, 37 °C). Cell adherence was observed under a microscope after 2 days and medium was changed to remove the cells without adherence. The eye-widening tissues were provided by the departments of burn plastic, Nanjing Drum Tower Hospital and fibroblasts were cultured according to above procedures.

### Surface marker expressions of UC-MSCs and fibroblasts by flow cytometry (FCM)

The FCM (BD, USA) analysis was performed to assay the surface marker expressions of UC-MSCs and fibroblasts. The cells in 100 μL PBS (Hyclone, SH.30256.01, China) were incubated with anti-CD14 (BD, 555397, USA), CD19(BD, 555412, USA),CD34(BD, 555821, USA), CD45(BD, 560976, USA), CD73(BD, 550257, USA), CD90(BD, 555596, USA), CD105(BD, 560839, USA),and HLA-DR(BD, 555561, USA) antibodies conjugated with FITC or PE for 15 min in darkness. The cells were assayed by FCM and the original data were analyzed by Flowjo (V10).

### Osteogenic, adipogenic, and chondrogenic differentiations

UC-MSCs and fibroblasts at P4 were digested and seeded on a six-well plate. The osteogenic differentiation and adipogenic differentiation mediums (Gibco, USA) were changed every 3 days to induce osteogenic and adipogenic differentiation, respectively. After 21 days, Alizarin red-S staining (Sigma-Aldrich, St. Louis, MO, USA) and Oil red O staining (Sigma-Aldrich, St. Louis, MO, USA) were used to stain the differentiated cells. Images were taken with a microscope (Olympus, Japan) and analyzed by Image J 1.52 software(National Institutes of Health, USA).The data were analyzed and visualized by GraphPad (Prism 5).

Cells were centrifuged at 200 g for 10 min and the supernatant was removed. Chondrogenic differentiation induction solution (Gibco, USA) was added into the experimental groups. The blank group was performed using complete medium. Then samples were placed in incubator (37 °C, 5% CO_2_) and medium was replaced every 3 days. On the 21st day, the samples were fixed by 4% paraformaldehyde for 1 h and O.C.T (SAKURA, USA) embedded sections were performed. After alcian blue staining, the images were observed under a microscope.

### Immunofluorescence

Cells were spread into 12-well plates on glass cover-slides. When cell confluence reached to 70%, the supernatant was discarded and 4% paraformaldehyde was fixed for half an hour. Primary antibodies of α-SMA (Abcam, ab32575), S100A4 (Abcam, ab124805), and vimentin (Abcam, ab92547) were incubated at 4 °C overnight. Cells were washed for 3 times with PBS on the next day and incubated with corresponding second antibody at room temperature for 1 h. Cell slides were collected and sealed by DAPI solution (Abcam, ab104139).The images were observed under a confocal microscope (Leica, Germany).

### Western blot

When cell confluence reached to 90%, UC-MSCs and fibroblasts were washed twice by PBS and then lysed using cold RIPA buffer (Beyotime P0013B) on ice for 15 min. Lysates of cells were cleared by centrifugation at 12,000 rpm for 25 min. Protein concentration of each sample was detected using the bicinchoninic acid assay (Beyotime, P0010). A total of 30 μg cell extract for each sample were resolved by SDS-PAGE, and then transferred to nitrocellulose membrane. The nitrocellulose membrane was probed with primary antibodies of α-SMA, S100A4, vimentin, and GAPDH (Service bio, GB11002). Final blots were detected using a supersensitive ECL system (Amersham).

### Immunoregulatory capability in vitro

UC-MSCs and fibroblasts were co-cultured with PBMCs in 6-well or 12-well plates. Specific procedures were carried out referring to our previous study [43] aiming to detect their regulatory effects on Th1, Th17, and Treg cell differentiations in vitro.

### RNA-seq

UC-MSCs and fibroblasts were digested for 3 min, then the cells were collected and counted as 2 × 10^6^ per sample (n = 3). A total of 1 mL Trizol (Thermo) was added into each sample and sent to company for RNA separation and library preparation.

RNA-seq libraries (Tru-seq, Illumina) were prepared from UC-MSCs and HFFs in 3 biological replicates. Total RNAs were extracted using the mirVana miRNA Isolation Kit (Ambion) and RNA integrity was evaluated using the Agilent 2100 Bioanalyzer (Agilent Technologies, Santa Clara, CA, USA). Samples with RNA Integrity Number (RIN) ≥ 7 were subjected to the subsequent analysis. Libraries were sequenced on the Illumina HiSeq sequencing platform and 125 bp/150 bp paired-end reads were generated.

Raw data were first filtered by Trimmomatic software and obtained clean reads were mapped to GRCh38.p12 version of Homo Sapiens reference genomes using HISAT2. FPKM value of each gene was calculated via cufflinks, and the read counts of each gene were obtained by htseq-count. Library construction and up-stream data processing of RNA-seq data above were conducted by OE Biotech Co., Ltd (Shanghai, China).

Principal Component Analysis (PCA) and Spearman correlation analyses of replicates in 2 groups were performed by TBtools software based on FPKM values. Differential gene expression analysis was conducted via the DESeq (2012) R package using the cutoff of |log2FoldChange|> 2, *q-value* < 0.01 based on BaseMean values which calculated by the same R package. Results of Gene Ontology (GO) and Kyoto Encyclopedia of Genes and Genomes (KEGG) enrichment analyses were obtained using online analysis tool Metascape after the input of certain gene sets. Protein–protein interaction network analysis was performed by another online tool STRING. In order to search for HFFs differentially expressed genes from another direction, we used a peak-calling tool, MACS2, to get significant clusters of HFFs reads, which was so-called peaks in this article, representing the high expression sites of HFFs replicates relative to the background UC-MSCs. The genome coordinates of those peaks of significance (*p* < 0.05) were then annotated by Homer software (hg38 genome). Only genes of those with significant peaks, significant clusters of mapped reads, and higher BaseMean level at the same time could be the most possible candidates of HFF-specific highly expressed genes for further analysis and research. We used Integrative Genomics Viewer (IGV) as the visual exploration tool for RNA-seq data.

### Metabolomic analysis

The culture medium was replaced with basal culture medium when cells grew to 80–90% confluence. After 24 h, the supernatant was collected and lyophilized by lyophilizer (Christ, EPSILON 2-6D LSC Plus). Samples (n = 6) were dissolved by methanol (containing 0.1% formic acid) before testing. UC-MSCs and HFFs were digested and collected after being washed twice by pre-cooled PBS and washed once by normal saline (0.9% sodium chloride solution). Before testing, 50 μL of water was added to each tube to re-suspend cells, then 450 μL methanol was added into tube. After swirling for 1 min, standing for 5 min, and centrifuging at room temperature for 15 min, supernatant was stored for analysis.

Samples were loaded through a UPLC system (Nexeraultra-high performance liquid chromatography LC-30A, Shimadzu) with an auto sampler. A HSS T3 column (1.8 μm, 2.1 mm ID × 100 mm, Waters) was used. The mobile phase A was water (containing 20 mM ammonium acetate and 20 mM ammonium hydroxide) and phase B was acetonitrile. The LC separations were 14 min per sample using the following scheme: (1) 0 min, 5% phase B; (2) 9 min, 95%phaseB; (3) 10 min, 95% phase B, (4) 11 min, 5% phase B, (5) 14 min, 5% phase B. All the changes were linear and the flow rate was 0.35 mL/min.MS analysis was performed using a Waters XEVO G2-XS QTof with a mimic multiple reaction monitoring (mMRM) mode. Typical operating parameters were set as follows: capillary voltage − 2.5 kV, source temperature 120 °C, desolvation temperature 350 °C, cone gas 50 L/h, desolvation gas 800 L/h. Both the cone and desolvation gases were nitrogen. The data were analyzed by MetaboAnalyst.

### Colitis model

After housed in SPF-level laboratory animal room, the 8-week-old female C57BL/6 mice (n = 8 per group) were fasted for 24 h, then were given enema (1:1 mixing of TNBS and anhydrous ethanol). Themodel mice were inverted for 3 min. After 6 h, model mice were treated with UC-MSCs and fibroblasts (1 × 10^6^/mouse) through intraperitoneal injection, respectively. Control group was treated with equal volume of PBS. The survival and body weight were observed daily for five consecutive days. At day 5 after cell therapy, mice were euthanized. Colons were harvested and MPO was detected using the MPO kit (Nanjing Jiancheng, A044-1–1, China). The data were analyzed and visualized by GraphPad.

### Hepatic fibrosis model

Female C57BL/6 mouse (8-week-old) was intraperitoneal administrated with 150 μL mixture of corn oil and CCL_4_ (corn oil: CCL_4_ = 3:1) (n = 8 per group), twice a week during an eight-week period. Sham group was administrated with equal volume of PBS, after eight weeks, mouse abdominal cavity was opened to confirm the success of constructing hepatic fibrosis model and orthotopic transplantation of UC-MSCs and HFFs (5 × 10^5^ cells/mouse) was carried through our previously reported novel minimally invasive open-flow microperfusion (OFM) technique [44]. Mice in control group were administrated with equal volume of PBS. After another 3 weeks, the orbital blood samples of mice were collected and centrifuged at 3500 rpm for 8 min to obtain the serum for glutamic-pyruvic transaminase (ALT) detection. After orbital blood collection, the mice were euthanized and the liver tissues of mice were harvested and homogenized to detect procollagen type III (PCIII) and laminin (LN) by ELASE kits (Nanjing Jiancheng) according to the instructions of Kits.

### Histopathology assays

Mice liver tissues were formalin-fixed and paraffin-embedded for hematoxylin and eosin (H&E) staining. Liver slices were fixed by 95% ethanol for 2-5 min after being washed with running water for 1 min.The slices were performed hematoxylin staining for 2–4 min and hydrochloric acid alcohol differentiation solution staining for 7 s. Then slices were put into ammonia water for 30 s and put into eosin solution for 7–10 s, then dehydrated by alcohol and permeabilized by xylene. After H&E staining, the slices were sealed by neutral resin for storage and observation.

For Masson staining, liver tissue slices firstly were dewaxed by xylene and alcohol and then corresponding steps were carried out according to the instructions of masson staining kit (Baso, BA4079A). Briefly, slices were treated by 1% glacial acetic acid for 1 min after staining and then dehydrated by alcohol and permeabilized by xylene. After sealed by neutral resin, images were captured by microscope.

For immunohistochemistry, the dewaxed liver sections were incubated with 3% hydrogen peroxide at room temperature for 15 min to remove endogenous peroxidase. After antigen retrieval, sections were blocked using 5% FBS at room temperature for 60 min and then incubated with primary α-SMA or collagen I antibody solution in a wet box at 4 °C overnight. The corresponding second antibody solution was incubated for 10 min at room temperature after washing with PBST. A volume of 50–100 μL DAB (Maxim, 2031) working solution was dropped onto the slices and color development was stopped in time by water under microscopic observation. After hematoxylin re-dying for 1–5 min, slices were dehydrated by alcohol and permeabilizedby xylene. Images were captured by microscope.

### Q-PCR

Total RNAs of liver tissue samples were extracted using Trizol reagent. The concentration of RNAs was measured by nanodrop (Thermo). Reverse transcription kit (Vazyme, R323-01) was used for reverse transcription according to instructions. 2 μL cDNA, 10 μL mix, 0.4 μL upstream primers, 0.4 μL downstream primers, and 7.2 μL water were mixed and added to the 96-well plate per well. The original Q-PCR results, expressed as the number of cycles, were converted into relative expression data using delta-Delta Ct method. Q-PCR data were collected by QuantStudio™ real-time PCR software v1.1. The primers of GAPDH, TGF-β, α-SMA, collagen I, MMP9, and TIMP1were listed as below.

GAPDH forward primer (TCGTCCCGTAGACAAAATGG), reverse primer (GAGGTCAAT GAAGGGGTCGT), TGF-β forward primer (GGACTCTCCACCTGCAAGAC), reverse primer (CATAGATGGCGTTGTTGCGG), α-SMA forward primer (GAACACGGCATCATCACCAAC), reverse primer (CTCCAGAGTCCAGCACAATACC), collagen I forward primer (GCTC CTCTTAGGGGCCACT), reverse primer (CCACGTCTCACCATTGGGG), MMP9 forward primer (CGCCTTGGTGTAGCACAACA), reverse primer (ACAGGGTTTGCCTTCTCCGTT), TIMP1forward primer (CGAGACCACCTTATACCAGCG), reverse primer (ATGACTGGGG TGTAGGCGTA).

### Immunoregulation assays in vivo

Spleens and mesenteric lymph nodes of mice were harvested and filtered after grinding, thensediments were re-suspended by 1 mL PBS after centrifugation. A total of 50 μL tissue homogenate were added to each well in 24-well plate and incubated with 500 μL 1640 culture medium at 37 °C for 5 h for detecting the Th1/Th17 and Tregs subsets according to above in vitro method.

After euthanizing the mice, we harvested the intestinal tracts from anus to cecum and then cleaned them 4—5 times with PBS after removing faeces. The intestines were expanded and divided into several sections as the size of soybean grains. Then, the intestines were transferred into a 50 ml centrifuge tube. Predigested liquid with RPMI1640 (Gibco, 61870036), 5 mM EDTA (Biosharp, BS107-100 g), 10 mM HEPES (Biosharp, BS106-100 g) were added to the tube. The shaker was used for digesting (37 °C, 150 rpm, 15 min). After filtering the liquid with to collect the tissues, the remaining intestinal tissues were cut into pieces and then added digestive solution containing RPMI1640, 1 mg/ml DNase (Roche, 10104159001), 1 g/ml mixed enzyme (Roche, 10269638001) at 37 °C, 150 rpm, 40 min. After filtrating with 100-mesh sieves, the cells centrifuged at 1500r for 5 min were lamina propria cells.

Cells were re-suspended by 400 μL PBS, we put 100 μL into a tube for FCM. CD4 + T cells were stained referring to the above methods of Th1, Th17, and Treg. Neutrophils were stained with CD45 (eBioscience, 17–0451-82), CD11b (eBioscience, 45–0112-82), Gr1 (BD, 553128) for 30 min at room temperature, then washed and put on the machine. Macrophages were stained with CD11b and F4/80 (eBioscience, 11–4801-85) for 30 min at room temperature first, then NOS2 (eBioscience, 12–5920-82) was used for 30 min at room temperature after fixing and permeabilizing.

### Statistical analysis

GraphPad Prism 5 Software (San Diego, CA, USA) was used for statistical processing. Quantitative data were expressed as Mean ± Standard Deviation (SD). The inter-group comparison was performed by one-way ANOVA. A *p* < 0.05 was considered significant difference.

## Supplementary Information

**Additional file 1: Fig. S1**. Cell numbers and phenotypic changes of immune cells in LP. (A) Immunoregulation of Th1 and Th17 in LP. (B) Immunoregulation of Treg in LP. (C) Changes of neutrophils in LP. (D) Phenotypic changes of macrophages in LP. (*p < 0.05, **p < 0.01, ***p < 0.001).

## Data Availability

The datasets used and/or analysed during the current study are available from the corresponding author on reasonable request.
