# Supplementary figures and images for: Human skin dermis-derived fibroblasts are a kind of functional mesenchymal stromal cells: judgements from surface markers, biological characteristics, to therapeutic efficacy
Source: Cell Biosci. 2022 Jul 12;12:105. doi: 10.1186/s13578-022-00842-2 (PMC9277801; doi:10.1186/s13578-022-00842-2)

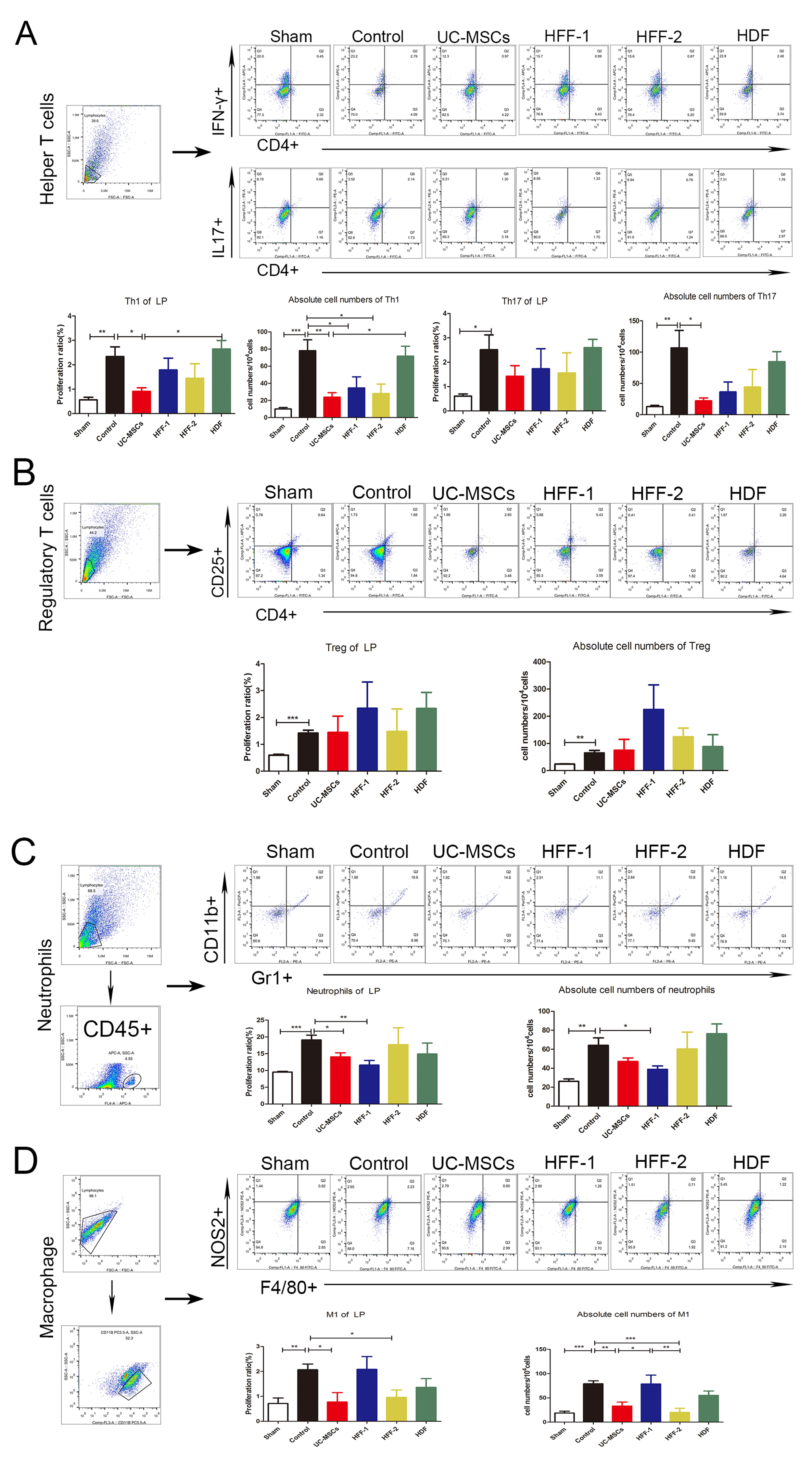

Supplement: Supplementary file 1 — Additional file 1: Fig. S1. Cell numbers and phenotypic changes of immune cells in LP. (A) Immunoregulation of Th1 and Th17 in LP. (B) Immunoregulation of Treg in LP. (C) Changes of neutrophils in LP. (D) Phenotypic changes of macrophages in LP. (*p < 0.05, **p < 0.01, ***p < 0.001). [file 13578_2022_842_MOESM1_ESM.tif]
